# Effect of Tomato Consumption on Liver Steatosis in MASLD: A Randomized Controlled Trial (POMOSANO Study)

**DOI:** 10.3390/nu18152476

**Published:** 2026-07-30

**Authors:** Rossella Tatoli, Rossella Donghia, Nicola Carella, Nicole Cerabino, Martina Di Chito, Marianna Zappimbulso, Pasqua Letizia Pesole, Dolores Stabile, Annalisa Ferro, Giovanni Pascale, Endrit Shahini, Francesca Pavone, Luigi Fontana, Gianluigi Giannelli

**Affiliations:** 1 Data Science Unit, National Institute of Gastroenterology IRCCS “Saverio de Bellis”, 70013 Bari, Italy; rossella.tatoli@irccsdebellis.it; 2 Clinical Research Unit, National Institute of Gastroenterology IRCCS “Saverio de Bellis”, 70013 Bari, Italy; nicola.carella@irccsdebellis.it (N.C.); francesca.pavone@irccsdebellis.it (F.P.); 3 Center of Nutrition for the Research and the Care of Obesity and Metabolic Diseases, National Institute of Gastroenterology IRCCS “Saverio de Bellis”, 70013 Bari, Italy; nicole.cerabino@irccsdebellis.it (N.C.); martina.dichito@irccsdebellis.it (M.D.C.); 4 Department of Gastroenterology, National Institute of Gastroenterology IRCCS “Saverio de Bellis”, 70013 Bari, Italy; marianna.zappimbulso@irccsdebellis.it (M.Z.); endrit.shahini@irccsdebellis.it (E.S.); 5 Core Facility Biobank, National Institute of Gastroenterology IRCCS “Saverio de Bellis”, 70013 Bari, Italy; letizia.pesole@irccsdebellis.it (P.L.P.); dolores.stabile@irccsdebellis.it (D.S.); 6 Laboratory of Clinical Pathology, National Institute of Gastroenterology IRCCS “Saverio de Bellis”, 70013 Bari, Italy; annalisa.ferro@irccsdebellis.it; 7 Radiology Unit, National Institute of Gastroenterology IRCCS “Saverio de Bellis”, 70013 Bari, Italy; giovanni.pascale@irccsdebellis.it; 8 Charles Perkins Centre, Faculty of Medicine and Health, The University of Sydney, Sydney, NSW 2006, Australia; luigi.fontana@sydney.edu.au; 9 Department of Endocrinology, Royal Prince Alfred Hospital, Sydney, NSW 2050, Australia; 10 Scientific Direction, National Institute of Gastroenterology IRCCS “Saverio de Bellis”, 70013 Bari, Italy; gianluigi.giannelli@irccsdebellis.it

**Keywords:** MASLD, liver steatosis, tomatoes, lycopene, randomized controlled trial, Mediterranean diet

## Abstract

**Background**: Metabolic dysfunction-associated steatotic liver disease (MASLD) is a common chronic liver disease for which lifestyle modification remains the main treatment. However, the role of specific dietary components is not fully understood. We investigated whether daily tomato consumption, a source of Mediterranean diet bioactive compounds, improves hepatic steatosis in adults with MASLD. **Methods**: In the randomized controlled POMOSANO trial, 79 adults with MASLD (BMI ≤ 30 kg/m^2^) were assigned to a tomato intervention group (*n* = 42), consuming 200 g/day of raw tomatoes and 50 g/day of tomato sauce, or to a control group (*n* = 37) following a tomato-free diet. The intervention lasted 6 weeks. Hepatic steatosis was assessed by the FibroScan^®^ controlled attenuation parameter (CAP). Secondary outcomes included anthropometric measures, body composition, liver stiffness, and metabolic and biochemical markers. Linear mixed-effects models were used to evaluate changes over time. **Results**: CAP decreased in both groups, with a greater reduction in the intervention group (median change: −35.00 vs. −18.00 dB/m). The adjusted linear mixed-effects model confirmed that the change in CAP in the control group was β = −12.81 (95% CI −25.55 to −0.08; *p* = 0.0490). Crucially, the treatment-by-time interaction was significant (β = −26.63, 95% CI −44.00 to −9.25; *p* = 0.0030), demonstrating a significantly greater reduction in hepatic steatosis in the tomato intervention group compared to the control group. CAP values were also significantly lower in the intervention group at follow-up (*p* = 0.0433). The adjusted linear mixed-effects model confirmed a significant reduction in CAP over time (β = −12.81, 95% CI −25.55 to −0.08; *p* = 0.0490) and a significant treatment-by-time interaction (β = −26.63, 95% CI −44.00 to −9.25; *p* = 0.0030), indicating a greater reduction in hepatic steatosis in the intervention group. No significant between-group differences were observed for liver stiffness, FIB-4 index, body weight, body composition, or most metabolic and biochemical markers. **Conclusions**: Daily consumption of raw tomatoes and tomato sauce reduced hepatic steatosis in adults with MASLD, supporting tomato-rich dietary strategies as a potential nutritional approach for MASLD management.

## 1. Introduction

Metabolic dysfunction-associated steatotic liver disease (MASLD) is the most common chronic liver disease worldwide and is closely associated with obesity, insulin resistance, and cardiometabolic dysfunction [1]. Excess hepatic fat accumulation and the resulting oxidative and inflammatory stress contribute to disease progression toward fibrosis, cirrhosis, and hepatocellular carcinoma [2]. Lifestyle intervention remains the cornerstone of MASLD management, with dietary modification representing a central therapeutic strategy [3].

The Mediterranean diet has consistently been associated with lower liver fat accumulation and improved metabolic health in individuals with MASLD [4,5]. However, most nutritional recommendations are based on overall dietary patterns and weight reduction, whereas the contribution of specific foods within the Mediterranean diet remains less clearly defined. Identifying foods capable of modulating hepatic steatosis independently of major weight loss could help refine nutritional approaches for MASLD management.

Tomatoes are a characteristic component of the Mediterranean dietary pattern and a major source of carotenoids, particularly lycopene, together with other antioxidant and anti-inflammatory phytochemicals [6,7]. The bioavailability of these compounds varies according to food preparation, with lycopene being more bioavailable in cooked tomato products [6]. Experimental studies suggest that tomato-derived compounds may influence oxidative stress, inflammatory signaling, and hepatic lipid metabolism, which are key mechanisms involved in MASLD pathogenesis [8,9]. Observational evidence has also shown inverse associations between tomato consumption and fatty liver prevalence [10]. However, evidence from randomized controlled trials evaluating the effects of tomato consumption on liver steatosis in adults with MASLD remains limited.

Therefore, the aim of the present randomized controlled trial was to evaluate whether daily consumption of raw tomatoes and tomato sauce reduces liver steatosis, assessed by a controlled attenuation parameter (CAP), in lean and overweight adults with MASLD.

## 2. Materials and Methods

### 2.1. Study Design

This was a randomized controlled nutritional intervention conducted in adults with metabolic dysfunction-associated steatotic liver disease (MASLD). The intervention lasted 6 weeks and compared daily tomato consumption with a tomato-free control diet. The study was approved by the Ethics Committee of the IRCCS Oncological Hospital “Giovanni Paolo II”, Bari, Italy (approval no. 238, 10 April 2024), and was conducted in accordance with the Declaration of Helsinki. The trial was registered at ClinicalTrials.gov (NCT06389851). This randomized controlled trial was reported in accordance with the Consolidated Standards of Reporting Trials (CONSORT) 2010 statement [11].

### 2.2. Participants

Participants were recruited during a liver disease prevention initiative through an online screening questionnaire. Individuals reporting hepatic steatosis were invited for further clinical evaluation.

Eligible participants were adults aged 18–65 years with metabolic dysfunction-associated steatotic liver disease (MASLD), diagnosed according to the criteria proposed by Rinella et al. Specifically, participants were required to have evidence of hepatic steatosis, together with at least one cardiometabolic risk factor. Harmful alcohol consumption (>30 g/day for men and >20 g/day for women) was excluded, as were other causes of chronic liver disease, including viral hepatitis, autoimmune liver diseases, and drug-induced liver injury [12].

**Figure 1:**
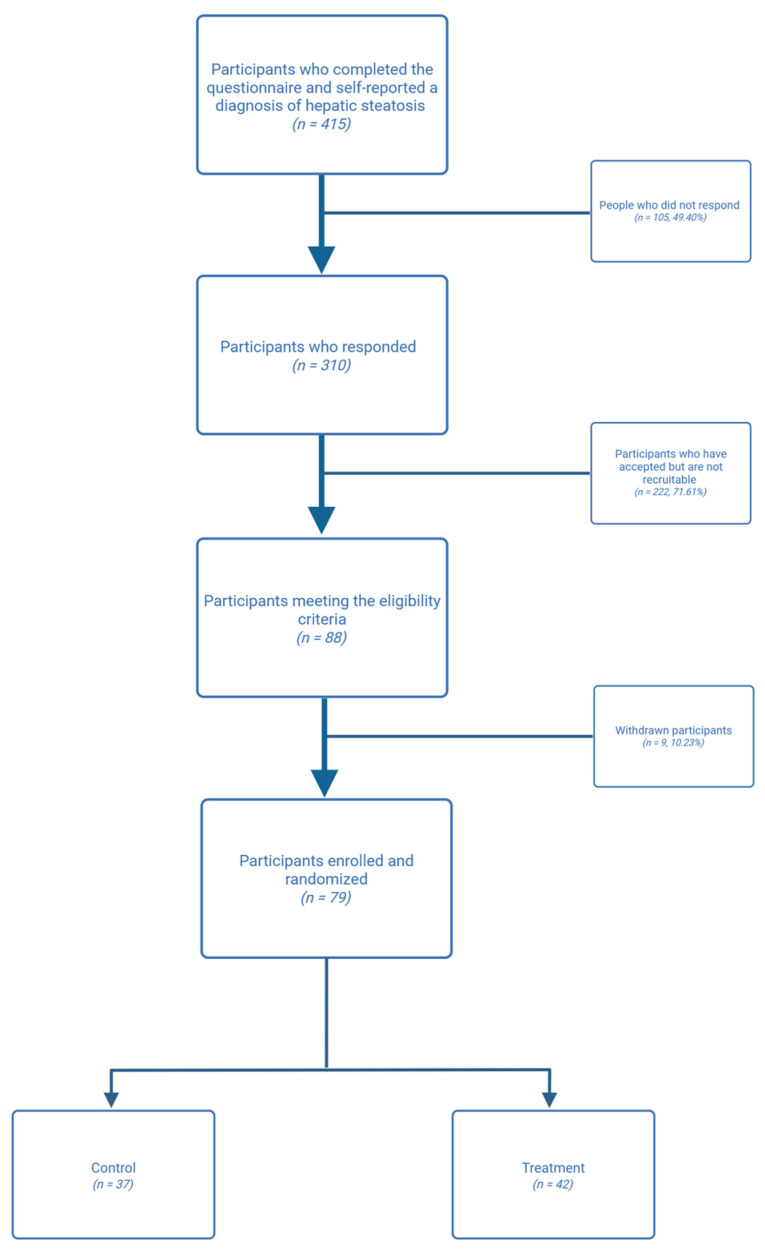
CONSORT flow diagram.

Hepatic steatosis was confirmed by transient elastography (FibroScan^®^, Echosens, Paris, France) using a controlled attenuation parameter (CAP) value of ≥275 dB/m. Participants with a body mass index (BMI) > 30 kg/m^2^ or evidence of other causes of chronic liver disease were excluded. Of 415 individuals completing the screening questionnaire, 79 eligible participants provided written informed consent and were randomized: 37 to the control group and 42 to the tomato intervention group. Participant flow is shown in Figure 1.

### 2.3. Randomization and Intervention

After completion of the baseline assessment and confirmation of eligibility, participants were randomized in a 1:1 allocation ratio using a computer-generated randomization (StataCorp LLC, College Station, TX, USA) sequence prepared and securely maintained by an independent biostatistician who was not involved in participant recruitment, eligibility assessment, or outcome evaluation. Investigators responsible for enrollment had no access to the allocation sequence before assignment, thereby ensuring allocation concealment. The randomization list consisted of a pre-established sequence of treatment assignments (control or tomato intervention), and participants were allocated sequentially according to the order of enrolment. A total of 80 allocations were generated before the start of recruitment, ensuring the planned allocation ratio throughout the enrolment period. The intervention group consumed 200 g/day of raw tomatoes and 50 g/day of tomato sauce for 6 weeks. The control group followed a tomato-free diet for the same period. All participants received standardized dietary instructions according to group allocation and were asked to maintain their usual lifestyle habits throughout the study.

### 2.4. Tomato Products

Tomatoes and tomato sauce were supplied by Azienda Agricola Lapietra (Monopoli, Italy), a certified producer using standardized hydroponic greenhouse cultivation. Production was conducted under controlled conditions with traceable quality procedures, water recycling, and energy recovery systems. This approach ensured consistency of the tomato products used throughout the intervention. Both raw tomatoes and tomato sauce were included to provide exposure to tomato-derived bioactive compounds in complementary forms, given the different bioavailability profiles of nutrients and phytochemicals in fresh and processed tomato products. The tomatoes were nickel-free and produced in compliance with Global GAP standards.

### 2.5. Clinical and Anthropometric Assessment

Clinical assessments were performed at baseline and after 6 weeks. Body weight, height, waist circumference, and neck circumference were measured using standardized procedures after an overnight fast. BMI was calculated as weight divided by height squared (kg/m^2^).

### 2.6. Body Composition

Body composition was assessed using bioelectrical impedance analysis and dual-energy X-ray absorptiometry (DXA). DXA scans were performed using a Hologic Horizon^®^ Wi densitometer (302801M) (Hologic, Inc., 600 Technology Drive, Newark, DE, USA) by trained technicians following standard calibration procedures.

### 2.7. Biochemical Analyses

Blood samples were collected between 8:00 a.m. and 9:00 a.m., after overnight fasting. Glucose, insulin, triglycerides, total cholesterol, LDL-cholesterol, HDL-cholesterol, AST, ALT, gamma-GT, and CRP concentrations were measured by a COBAS 8000 autoanalyzer (ROCHE Diagnostic SPA, Monza, Italy). HbA1c was determined by the automatic system for capillary electrophoresis Capillarys 3 OCTA (Sebia Italia S.r.l., Bagno a Ripoli, Firenze, Italy).

Biochemical, metabolic, hepatic, lipid, inflammatory, hormonal, and hematological parameters were assessed by qualified laboratory personnel in compliance with institutional guidelines and good laboratory practice, ensuring the accuracy and reproducibility of the results. Laboratory staff were blinded to treatment allocation throughout the analytical process.

### 2.8. Liver Assessment

Hepatic steatosis and liver stiffness were assessed by transient elastography (FibroScan^®^, Echosens, Paris, France) after a minimum 4 h fast. Steatosis was quantified using the controlled attenuation parameter (CAP, dB/m), and liver stiffness was expressed in kPa. All measurements were performed by trained operators blinded to treatment allocation.

Transient elastography was performed using the M or L probe, according to the manufacturer’s recommendations. Only tests with at least 10 valid measurements and meeting standard reliability criteria (IQR/median ≤ 30%) were considered valid. Where possible, follow-up assessments were performed using the same probe and by the same trained operator, who remained blinded to treatment assignment.

The Fatty Liver Index (FLI) was calculated according to the equation developed by Bedogni et al., using body mass index (BMI), waist circumference, serum triglycerides, and gamma-glutamyl transferase (GGT) levels: FLI = [e^0.953^ × log (Triglycerides) + 0.139 × BMI + 0.718 × log (GGT) + 0.053 × Waist Circumference − 15.745)]/[1 + e^0.953^ × log (Triglycerides) + 0.139 × BMI + 0.718 × log (GGT) + 0.053 × Waist Circumference − 15.745)] × 100 [13].

### 2.9. Statistical Analysis

Given the absence of previous randomized controlled trials evaluating the effect of tomato consumption on CAP in adults with MASLD, reliable estimates of the expected treatment effect and its variability were unavailable. Therefore, this study was designed as an exploratory randomized controlled trial. A pragmatic sample size of approximately 30 participants per group was considered feasible and adequate to provide preliminary estimates of treatment effect and variance, with the primary aim of informing the design of future adequately powered confirmatory studies, rather than formally testing a predefined hypothesis.

Model assumptions for linear mixed-effects models were evaluated through residual diagnostics. Normality and homoscedasticity of conditional residuals were assessed using visual inspection of Q-Q plots and residual vs. fitted value plots, supplemented by formal normality tests on residuals. To evaluate changes in CAP over time, a linear mixed-effects model was fitted with CAP as the dependent variable. Treatment group, time, and the treatment-by-time interaction were included as fixed effects. The model was adjusted for age, sex, and BMI, with participant-specific random intercepts to account for repeated measurements. Results are reported as β coefficients with 95% confidence intervals.

The distribution of continuous variables was assessed using the Shapiro–Wilk test. Since several variables were not normally distributed, continuous data are reported as median and interquartile range (IQR), and categorical variables as frequency and percentage (%). Baseline characteristics were compared between groups using the Wilcoxon rank-sum test for continuous variables and the chi-square or Fisher’s exact test for categorical variables, as appropriate. Within-group changes from baseline to follow-up were assessed using the Wilcoxon signed-rank test for continuous variables and McNemar or McNemar-Bowker tests for categorical variables. Between-group comparisons at follow-up were performed using the Wilcoxon rank-sum test or Fisher’s exact test, as appropriate.

All tests were two-tailed, and statistical significance was set at *p* < 0.05. Statistical analyses were performed using Stata version 19 (StataCorp LLC, College Station, TX, USA), and plots were generated using RStudio version 2026.01 (“Apple Blossom” Release; Posit Software, PBC, Boston, MA, USA).

## 3. Results

### 3.1. Study Participants

A total of 79 participants were randomized, including 37 in the control group and 42 in the tomato intervention group. Baseline demographic and socioeconomic characteristics are summarized in Table 1. Median age was 59.4 (IQR 14.91) years in the control group and 56.7 (IQR 14.67) years in the intervention group. Most participants were male (67.6% and 73.8%, respectively), and the prevalence of current smoking was low in both groups. No statistically significant differences were observed in baseline demographic or socioeconomic characteristics between the control and intervention groups, indicating that the two groups were well balanced at study entry.

### 3.2. Anthropometric and Body Composition Measurements

Changes in anthropometric and DXA-derived body composition parameters are summarized in Table 2. Body weight, BMI, waist circumference, total body fat, fat mass/height^2^, and DXA-derived visceral adiposity estimates remained largely unchanged during the intervention, with no significant between-group differences observed at follow-up. Bioelectrical impedance parameters are reported in Supplementary Table S1.

### 3.3. Liver-Related Outcomes

**Figure 2:**
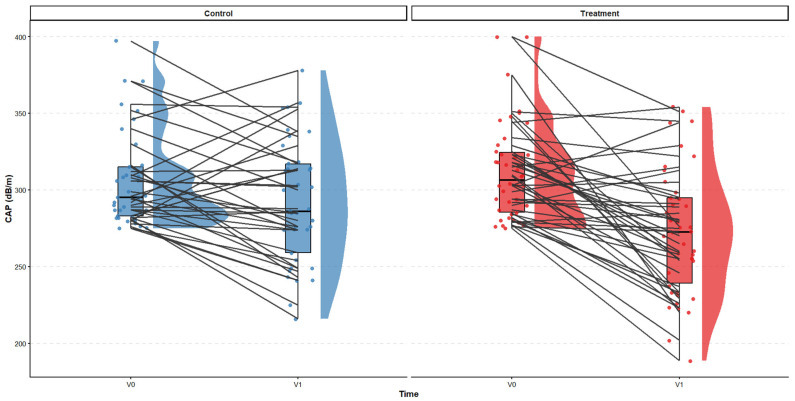
Change in CAP values from baseline to 6 weeks by study group. Half-violin boxplots show the distribution of controlled attenuation parameter (CAP) values at baseline (V0) and after 6 weeks (V1) in the control and tomato intervention groups. Individual points connected by lines represent within-participant changes over time.

Liver-related outcomes are summarized in Table 3 and Figure 2. CAP decreased in both groups, but the reduction was greater in the intervention group. In the control group, CAP decreased from 295.00 (IQR 32.00) to 286.0 (IQR 58.00) dB/m (median change −18.00 [IQR 37.00]; *p* = 0.0080). In the intervention group, CAP decreased from 306.50 (IQR 40.00) to 272.50 (IQR 58.00) dB/m (median change −35.00 [IQR 59.00]; *p* < 0.0001). The between-group comparison favored the intervention group (*p* = 0.0433).

Figure 2 illustrates a downward shift in the distribution of CAP values in the intervention group at follow-up, together with a consistent pattern of individual reductions over time.

In the adjusted linear mixed-effects model (Table 4), no significant baseline difference in CAP was observed between groups (β = 5.38, 95% CI −10.22 to 20.98; *p* = 0.4999), with the control group as the baseline reference.

In the adjusted linear mixed-effects model (Table 4), no significant baseline difference in CAP was observed between groups (β = 5.38, 95% CI −10.22 to 20.98; *p* = 0.4999, with the control group as baseline reference). The adjusted change in CAP over time for the control group was β = −12.81 (95% CI −25.55 to −0.08; *p* = 0.0490). The primary treatment effect estimate, represented by the treatment-by-time interaction term, was statistically significant (β = −26.63, 95% CI −44.00 to −9.25; *p* = 0.0030), confirming a greater reduction in CAP over time in the tomato intervention group relative to control.

The adjusted marginal means derived from the mixed-effects model are illustrated in Supplementary Figure S1, showing a greater decline in predicted CAP values over time in the tomato intervention group compared with the control group. No significant changes were observed in liver stiffness, FIB-4 index, or FIB-4 categorical risk stratification during the intervention period.

### 3.4. Biochemical Outcomes

No significant between-group differences were observed for glucose metabolism, lipid profile, inflammatory markers, liver enzymes, or haematological parameters after 6 weeks of intervention (Table 5 and Supplementary Table S2).

## 4. Discussion

In this randomized controlled trial, daily consumption of raw tomatoes and tomato sauce for 6 weeks resulted in a greater reduction in CAP-defined hepatic steatosis in adults with MASLD compared with a tomato-free control diet. This effect remained significant after adjustment for age, sex, and BMI and occurred without substantial changes in body weight, DXA-derived total or regional adiposity, glucose metabolism, lipid profile, inflammatory markers, liver stiffness, or fibrosis indices. These findings suggest that tomato consumption may reduce hepatic fat accumulation independently of major changes in body weight, total and abdominal adiposity.

The present study adds to the limited clinical evidence evaluating specific whole foods in MASLD. While nutritional recommendations largely focus on dietary patterns and weight reduction, controlled data examining the contribution of individual foods remain scarce [3]. In this context, tomatoes represent a biologically plausible intervention because they are a key component of the Mediterranean diet and provide lycopene, β-carotene, polyphenols, and other antioxidant and anti-inflammatory compounds [6,7]. Previous observational data suggested an inverse association between tomato intake and fatty liver disease [8], but randomized evidence in adults with MASLD remains limited.

Several mechanisms may plausibly contribute to the observed reduction in CAP. Tomato-derived bioactive compounds have been shown to modulate oxidative stress, inflammatory signaling, mitochondrial function, and hepatic lipid metabolism [14,15,16]. Tomato-based interventions have also been associated with lower circulating concentrations of IL-6 and TNF-α in overweight and obese individuals [17].

Carotenoids such as lycopene and β-carotene may also influence hepatic lipid accumulation and insulin sensitivity [18,19]. In experimental models, lycopene-rich tomato supplementation reduced hepatic steatosis and inflammation through effects on lipid oxidation, lipogenesis, adiponectin signaling, and liver–adipose tissue crosstalk [20,21]. In their experiments, Pipitone et al. found an improvement in body weight and liver steatosis after a high-fat diet supplemented with red tomatoes [21]. They hypothesized that this effect is attributable to the β-carotene content, which reduces the accumulation of lipids in the liver [20]. Lycopene is another main pigment of tomatoes and is responsible for their characteristic red color [21]. It is known for its anti-inflammatory and antioxidant properties, which vary depending on bioavailability, metabolism, isomerization, and interactions with other nutritional components [22]. In raw cherry tomatoes, lycopene is present in its trans isoform; heat treatments, such as cooking, increase its bioavailability by promoting isomerization from the trans to the cis isoform [6]. Being a fat-soluble substance, lycopene increases its bioavailability when combined with dietary fat sources [23], for example, extra virgin olive oil, commonly used as a dressing for tomatoes in the Mediterranean basin.

Some studies have demonstrated the protective effect of a diet supplemented with tomatoes rich in lycopene against the risk of developing certain chronic diseases [24]. Furthermore, it has been observed that, compared to the healthy population, individuals with hepatic steatosis have lower circulating levels of lycopene, highlighting the possible role of this pigment in the development of steatosis [25]. Studies conducted on obese mice have shown that lycopene is preferentially metabolized into apolycopenoids, which have been shown to alleviate diet-induced steatosis and liver inflammation [20]. In particular, apo-10’-lycopenoic acid induces sirtuin 1 (SIRT1), a key enzyme in the regulation of energy homeostasis, lipid metabolism, and inflammatory response [26]. In the liver, SIRT1 is required for AMPK activation [27], and the activation of the SIRT1-AMPK axis has been identified as a strategy for the treatment of hepatic steatosis in MASLD. Upregulation of SIRT1 activity may be one of the mechanisms through which a tomato-enriched diet reduces hepatic steatosis and inflammation. This concept is supported by the results of Cheng-Chung Li et al. In their study, they demonstrate how a diet supplemented with tomato powder alleviates the severity of liver steatosis and modulates the inflammatory response in the liver and mesenteric adipose tissue (MAT) induced by the HFD in BCO1−/−BCO2−/− double KO mice [28]. Improved inflammation levels in the MAT also have a positive effect on the liver thanks to increased adiponectin levels. In the liver, adiponectin can activate the transcription of PPARα, a receptor involved in energy and lipid homeostasis [29,30]. Several studies have identified an inverse correlation between circulating adiponectin levels and liver steatosis grade [31]. The increased expression of adiponectin in MAT in response to dietary supplementation with tomato could therefore be considered a therapeutic target/tool for MASLD.

Four potential mechanisms through which a tomato-enriched diet can improve MASLD and, in particular, liver steatosis can be identified: reduction in de novo fatty acid synthesis, increase in fatty acid oxidation, reduction in fatty acid uptake by the liver, and improvement in the inflammatory state.

In our study, the combined use of raw tomatoes and tomato sauce is another relevant aspect of the intervention. Tomato processing increases lycopene bioavailability by promoting isomerization from the trans to the cis form, whereas raw tomatoes provide heat-sensitive micronutrients and phytochemicals [6,22,23]. Thus, the intervention may have provided complementary exposures to tomato-derived compounds with different absorption profiles.

No significant changes were observed in liver stiffness or FIB-4. This is not unexpected given the short intervention duration and the low prevalence of advanced fibrosis in the study population. Similarly, the absence of major changes in circulating metabolic and inflammatory biomarkers suggests that the reduction in hepatic fat may precede detectable systemic metabolic changes or involve pathways not captured by routine biochemical testing. The modest reduction in CAP observed in the control group may reflect participation in a dietary intervention study, increased health awareness, regression to the mean, or unmeasured lifestyle changes during follow-up; however, the greater reduction in the intervention group and the significant treatment-by-time interaction support a treatment-related effect.

This study has several strengths, including its randomized controlled design, standardized tomato intervention, blinded transient elastography assessment, and comprehensive phenotyping. A major strength of the study is the use of DXA-derived body composition assessment, which enabled quantification of total and regional adiposity, including visceral fat estimates. This allowed us to demonstrate that the reduction in CAP occurred despite minimal changes in overall and abdominal adiposity. The reduction in CAP occurred despite minimal changes in these parameters, supporting the interpretation that the observed improvement in hepatic steatosis was not driven by major changes in overall or abdominal fat mass. Limitations include the modest sample size, short duration, reliance on dietary counselling and self-reported adherence, and absence of circulating lycopene or carotenoid measurements. The findings should therefore be considered hypothesis-generating and require confirmation in larger and longer-term trials.

## 5. Conclusions

Daily consumption of raw tomatoes and tomato sauce may represent a promising dietary strategy for reducing hepatic steatosis in adults with MASLD. These findings support the potential role of tomato-based interventions as part of nutritional management for MASLD and warrant confirmation in larger, adequately powered randomized controlled trials with longer follow-up and mechanistic investigations.

## Figures and Tables

**Table 1:** Baseline characteristics of the study population (n = 79).

Parameters *	p50 (IQR) or %		*p* ^^^
	Control (n = 37)	Treatment (n = 42)	
Age (yrs)	59.37 (14.91)	56.71 (14.67)	0.4030 ^†^
Gender (M) (%)	25 (67.57)	31 (73.81)	0.5420
Smoke (Yes) (%)	3 (8.11)	2 (4.76)	0.6610
Education (%)			0.1210
Elementary School	1 (2.70)	0 (0.00)	
Middle School	8 (21.62)	3 (7.14)	
High School	17 (45.95)	22 (52.38)	
Degree	10 (27.03)	17 (40.48)	
Post Degree	1 (2.70)	0 (0.00)	
Marital Status (%)			0.2700
Unmarried	3 (8.11)	6 (14.29)	
Married	31 (83.78)	35 (83.33)	
Separated	1 (2.70)	0 (0.00)	
Divorced	0 (0.00)	1 (2.38)	
Widow/er	2 (5.41)	0 (0.00)	
Job (%)			0.5830
Legislators, Entrepreneurs, and Senior Management	1 (2.70)	3 (7.14)	
Intellectual, Scientific, and Highly Specialized Professions	5 (13.51)	8 (19.05)	
Technical Professions	2 (5.41)	4 (9.52)	
Clerical and Office Work Professions	9 (24.32)	6 (14.29)	
Skilled Professions in Commerce and Services	4 (10.81)	2 (4.76)	
Craftsmen, Skilled Workers, and Farmers	1 (2.70)	4 (9.52)	
Plant Operators, Machine Operators, and Vehicle Drivers	2 (5.41)	0 (0.00)	
Unskilled Professions	3 (8.11)	4 (9.52)	
Armed Forces	1 (2.70)	3 (7.14)	
Retired	9 (24.32)	8 (19.05)	

* Values are reported as median (IQR) for continuous variables and n (%) for categorical variables. ^^^ Chi-square or Fisher test when necessary; ^†^ Wilcoxon rank-sum test.

**Table 2:** Changes in anthropometric and DXA parameters during the intervention.

Parameters *	Control Group (n = 37)	Intervention Group (n = 42)	Between-Group ^¥^
Body weight (kg)			
Baseline	77.80 (11.00)	79.50 (10.50)	
Change after 6 weeks	−0.35 (2.00)	−0.75 (2.60)	0.9662
BMI (kg/m^2^)			
Baseline	27.76 (2.56)	27.17 (3.85)	
Change after 6 weeks	−0.11 (0.73)	−0.24 (0.95)	0.2106
Waist circumference (cm)			
Baseline	97.0 (9.0)	97.0 (8.0)	
Change after 6 weeks	−1.0 (5.0)	−1.5 (3.0)	0.8735
Total body fat (%)			
Baseline	32.9 (12.40)	31.6 (6.70)	
Change after 6 weeks	−0.1 (1.75)	−0.3 (1.20)	0.3866
Fat mass/height^2^ (kg/m^2^)			
Baseline	8.55 (3.68)	8.63 (2.58)	
Change after 6 weeks	0.0 (0.54)	−0.1 (0.40)	0.3724
Estimated VAT mass (g)			
Baseline	852.0 (230.00)	930.5 (322.00)	
Change after 6 weeks	−0.5 (72.50)	10.0 (101.00)	0.2932
Estimated VAT area (cm^2^)			
Baseline	179.0 (49.00)	192.0 (67.00)	
Change after 6 weeks	0.00 (14.50)	2.00 (20.00)	0.3806

* Values are reported as median (IQR). ^¥^ Between-group comparisons were performed using the Wilcoxon rank-sum test. Abbreviations: BMI, body mass index; DXA, dual-energy X-ray absorptiometry; VAT, visceral adipose tissue.

**Table 3:** Changes in liver-related parameters during the intervention.

Parameters *	Control Group (n = 37)	Intervention Group (n = 42)	Between-Group ^¥^
CAP (dB/m)			
Baseline	295.00 (32.00)	306.50 (40.00)	
Change after 6 weeks	−18.00 (37.00)	−35.00 (59.00)	0.0433
FLI			
Baseline	55.35 (25.94)	47.82 (29.68)	
Change after 6 weeks	−1.66 (13.04)	−2.50 (12.79)	0.6007
Liver stiffness (kPa)			
Baseline	5.30 (1.80)	5.35 (2.20)	
Change after 6 weeks	−0.20 (1.60)	−0.05 (2.30)	0.7045
FIB-4 index			
Baseline	0.98 (0.58)	0.97 (0.49)	
Change after 6 weeks	0.03 (0.26)	0.05 (0.36)	0.5284
FIB-4 category, n (%)			
≤2.67 at baseline	37 (100.0)	41 (97.62)	
≤2.67 after 6 weeks	36 (97.30)	41 (97.62)	
>2.67 at baseline	0 (0.00)	1 (2.38)	
>2.67 after 6 weeks	1 (2.70)	1 (2.80)	0.9999 ^^^
FIB-4 risk category, n (%)			
<1.30 at baseline	28 (75.68)	34 (80.95)	
<1.30 after 6 weeks	28 (75.70)	29 (69.10)	
1.30–2.67 at baseline	9 (24.32)	7 (16.67)	
1.30–2.67 after 6 weeks	8 (21.60)	12 (28.60)	
>2.67 at baseline	0 (0.0)	1 (2.38)	
>2.67 after 6 weeks	1 (2.70)	1 (2.38)	0.8010 ^^^

* Values are reported as median (IQR) or n (%). ^¥^ Between-group comparisons were performed using the Wilcoxon rank-sum test for continuous variables and ^^^ Fisher’s exact test for categorical variables. Abbreviations: CAP, controlled attenuation parameter; FLI, Fatty Liver Index; FIB-4, Fibrosis-4 index.

**Table 4:** Mixed-effects regression model for changes in CAP values over time.

Variable	β	se (β)	*p*	95% C.I.
Treatment group	5.38	7.96	0.4999	−10.22 to 20.98
Time: after 6 weeks	−12.81	6.49	0.0490	−25.55 to −0.08
Treatment × time	−26.63	8.86	0.0030	−44.00 to −9.25

Values are β coefficients from a linear mixed-effects model adjusted for age, sex, and BMI, with participant-specific random intercepts. Reference categories: Control group for Treatment; Baseline (V0) for Time. The interaction term (Treatment\times Time) represents the difference-in-differences estimate between the intervention and control groups. Abbreviations: CAP, controlled attenuation parameter; β, Coefficient; se (β), Standard Error of β; 95% C.I., Confidence Interval at 95%.

**Table 5:** Changes in metabolic and hepatic biochemical parameters during the intervention.

Parameters *	Control Group (n = 37)	Intervention Group (n = 42)	Between-Group ^¥^
Glucose (mg/dL)			
Baseline	95.00 (14.00)	98.00 (19.00)	
Change after 6 weeks	3.00 (10.00)	1.50 (8.00)	0.7047
Insulin (µIU/mL)			
Baseline	12.00 (7.70)	14.10 (7.50)	
Change after 6 weeks	−0.20 (5.80)	−1.30 (6.70)	0.5235
HbA1c (%)			
Baseline	5.50 (0.40)	5.60 (0.50)	
Change after 6 weeks	0.00 (0.20)	0.00 (0.20)	0.4959
AST (U/L)			
Baseline	19.00 (6.00)	21.00 (10.00)	
Change after 6 weeks	0.00 (7.00)	0.00 (6.00)	0.2420
ALT (U/L)			
Baseline	21.00 (15.00)	27.50 (14.00)	
Change after 6 weeks	−2.00 (7.00)	−2.00 (7.00)	0.1498
GGT (U/L)			
Baseline	24.00 (22.00)	24.00 (25.00)	
Change after 6 weeks	−1.00 (9.00)	−0.50 (7.00)	0.8202
Total cholesterol (mg/dL)			
Baseline	183.00 (45.00)	183.00 (60.00)	
Change after 6 weeks	1.00 (19.00)	−1.00 (24.00)	0.6096
HDL cholesterol (mg/dL)			
Baseline	53.00 (17.00)	49.00 (20.00)	
Change after 6 weeks	1.00 (6.00)	−1.00 (8.00)	0.1980
LDL cholesterol (mg/dL)			
Baseline	112.00 (52.00)	108.50 (51.00)	
Change after 6 weeks	2.00 (15.00)	−2.00 (22.00)	0.5993
Triglycerides (mg/dL)			
Baseline	105.00 (77.00)	110.50 (46.00)	
Change after 6 weeks	−8.00 (28.00)	−0.50 (52.00)	0.5140
CRP (mg/dL)			
Baseline	0.15 (0.23)	0.11 (0.21)	
Change after 6 weeks	0.01 (0.08)	−0.01 (0.08)	0.4122

* Values are reported as median (IQR). ^¥^ Between-group comparisons were performed using the Wilcoxon rank-sum test. Abbreviations: HbA1c, glycated hemoglobin; AST, aspartate aminotransferase; ALT, alanine aminotransferase; GGT, gamma-glutamyl transferase; HDL, high-density lipoprotein; LDL, low-density lipoprotein; CRP, C-reactive protein.

## Data Availability

The original contributions presented in the study are included in the article. Further inquiries can be directed to the corresponding author.

## Supplementary Materials

The following supporting information can be downloaded at: https://www.mdpi.com/article/10.3390/nu18152476/s1, Table S1: Changes in bioelectrical impedance parameters during the intervention; Table S2 Additional biochemical and haematological parameters during the intervention; Figure S1: Adjusted marginal means of CAP values by treatment group over time. Adjusted marginal means with 95% confidence intervals derived from the mixed-effects regression model evaluating the interaction between treatment group and time on CAP values. Estimates were adjusted for age, sex, and BMI.
