# Supplementary material for: Effect of Tomato Consumption on Liver Steatosis in MASLD: A Randomized Controlled Trial (POMOSANO Study)
Source: Nutrients. 2026 Jul 30;18(15):2476. doi: 10.3390/nu18152476 (PMC13468337; doi:10.3390/nu18152476)
Supplement: Supplementary file 1 [file nutrients-18-02476-s001.zip › nutrients-4431408-supplementary.pdf]

nutrients

MDPI

Supplementary Materials

Table S1. Changes in bioelectrical impedance parameters during the intervention.

Parameters *	Control group (n=37)	Intervention group (n=42)	Between-group †
Phase angle			
Baseline	6.30 (1.10)	6.25 (1.00)	
Change after 6 weeks	−0.10 (0.60)	−0.10 (0.60)	0.6474
Body cell mass index			
Baseline	11.10 (2.80)	11.10 (2.00)	
Change after 6 weeks	0.10 (0.90)	0.05 (1.20)	0.9513
Body cell mass (kg)			
Baseline	32.60 (11.30)	32.70 (8.00)	
Change after 6 weeks	0.30 (2.30)	−0.10 (3.30)	0.7601
Fat-free mass (kg)			
Baseline	57.80 (13.90)	58.65 (10.50)	
Change after 6 weeks	0.90 (3.00)	0.00 (3.80)	0.5825
Fat mass (kg)			
Baseline	21.70 (8.50)	19.95 (5.20)	
Change after 6 weeks	−1.10 (2.90)	−1.20 (3.30)	0.7159
Total body water (L)			
Baseline	41.90 (10.00)	42.90 (8.70)	
Change after 6 weeks	0.60 (1.90)	−0.05 (3.20)	0.7453
Extracellular water (L)			
Baseline	18.60 (2.60)	19.05 (2.80)	
Change after 6 weeks	0.20 (1.70)	0.10 (1.70)	0.4325

* Values are reported as median (IQR).
† Between-group comparisons were performed using the Wilcoxon rank-sum test.

Table S2. Additional biochemical and haematological parameters during the intervention.

Parameters *	Control group (n=37)	Intervention group (n=42)	Between-group †
Creatinine (mg/dL)			
Baseline	0.90 (0.22)	0.92 (0.29)	
Change after 6 weeks	0.00 (0.07)	0.00 (0.09)	0.5958
eGFR (mL/min/1.73m²)			
Baseline	90.00 (17.00)	88.50 (23.00)	
Change after 6 weeks	−1.00 (6.00)	0.00 (7.00)	0.7451
Uric acid (mg/dL)			
Baseline	5.10 (1.60)	5.65 (1.50)	
Change after 6 weeks	0.00 (0.60)	−0.20 (0.90)	0.2067
Platelets (10^9/L)			
Baseline	250.00 (82.00)	254.50 (68.00)	
Change after 6 weeks	6.00 (25.00)	−8.50 (35.00)	0.3826
Total protein (g/dL)			
Baseline	7.10 (0.60)	7.15 (0.50)	
Change after 6 weeks	0.00 (0.40)	−0.15 (0.40)	0.3810
Sodium (mmol/L)			
Baseline	141.00 (3.00)	141.00 (3.00)	
Change after 6 weeks	−1.00 (4.00)	0.00 (2.00)	0.4767
Potassium (mmol/L)			
Baseline	4.50 (0.40)	4.40 (0.40)	
Change after 6 weeks	0.00 (0.40)	−0.10 (0.40)	0.2840
Calcium (mg/dL)			
Baseline	9.50 (0.70)	9.50 (0.60)	
Change after 6 weeks	0.00 (0.50)	−0.10 (0.50)	0.4238
Phosphorus (mg/dL)			
Baseline	3.30 (0.70)	3.30 (0.90)	
Change after 6 weeks	−0.10 (0.60)	−0.10 (0.50)	0.4254
Sideremia (µg/dL)			
Baseline	89.00 (32.00)	90.50 (48.00)	
Change after 6 weeks	4.00 (43.00)	0.00 (37.00)	0.2529
TSH (µIU/mL)			
Baseline	1.57 (1.02)	1.48 (1.29)	
Change after 6 weeks	−0.03 (0.50)	−0.03 (0.60)	0.4984
FT3 (pg/mL)			
Baseline	3.31 (0.51)	3.33 (0.66)	
Change after 6 weeks	0.05 (0.43)	−0.06 (0.51)	0.6654

(continue)

https://doi.org/10.3390/nu18152476

Parameters *	Control group (n=37)	Intervention group (n=42)	Between-group †
FT4 (ng/dL)			
Baseline	1.19 (0.16)	1.23 (0.28)	
Change after 6 weeks	0.00 (0.11)	−0.01 (0.23)	0.3851
NT-proBNP (pg/mL)			
Baseline	40.00 (42.00)	39.50 (31.00)	
Change after 6 weeks	5.00 (34.00)	−3.00 (39.00)	0.3216
Testosterone (ng/dL)			
Baseline	358.00 (438.00)	378.00 (464.10)	
Change after 6 weeks	−4.10 (52.20)	−7.05 (48.00)	0.9669
Prolactin (ng/mL)			
Baseline	7.30 (3.70)	7.95 (3.60)	
Change after 6 weeks	−0.40 (2.60)	−0.05 (2.60)	0.6869
Vitamin D (ng/mL)			
Baseline	22.90 (13.40)	23.35 (7.40)	
Change after 6 weeks	−0.20 (11.00)	2.05 (7.00)	0.7638
Ferritin (ng/mL)			
Baseline	184.00 (238.00)	163.00 (295.00)	
Change after 6 weeks	−4.00 (24.00)	1.50 (44.00)	0.4953
PTH (pg/mL)			
Baseline	33.30 (15.40)	37.20 (14.90)	
Change after 6 weeks	2.00 (11.60)	−0.20 (17.00)	0.6619

* Values are reported as median (IQR).
Abbreviations: eGFR, Estimated Glomerular Filtration Rate; TSH, Thyroid-Stimulating Hormone; FT3, Free Triiodothyronine; FT4, Free Thyroxine; NT-proBNP, N-terminal pro-B-type Natriuretic Peptide; PTH, Parathyroid Hormone.
† Between-group comparisons were performed using the Wilcoxon rank-sum test.

https://doi.org/10.3390/nu18152476

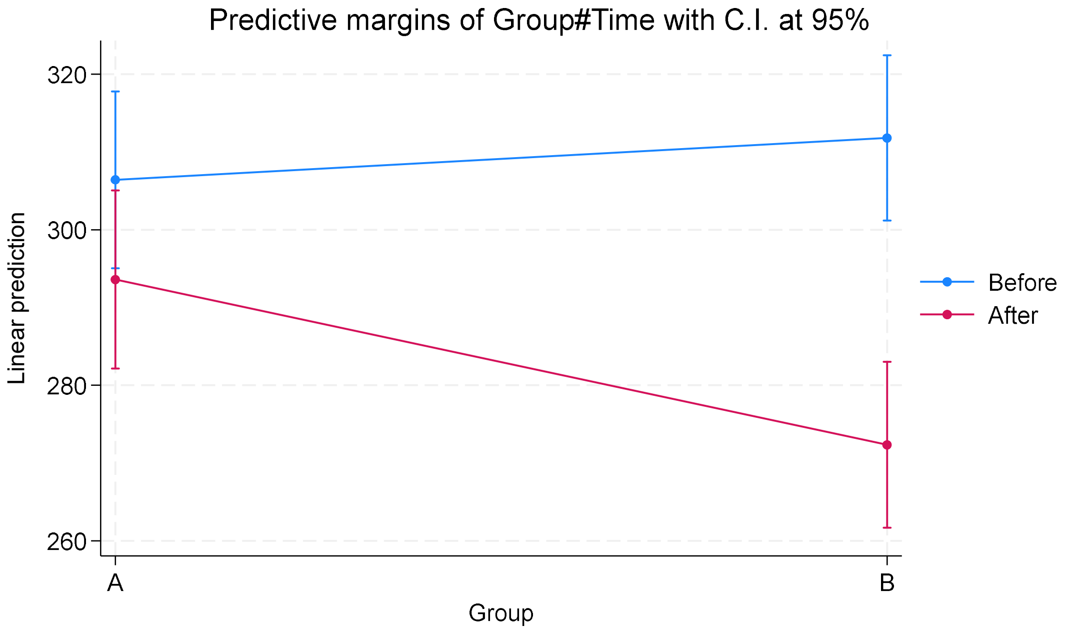

Figure S1. Adjusted marginal means of CAP values by treatment group over time.
Adjusted marginal means with 95% confidence intervals derived from the mixed-effects regression model evaluating the interaction between treatment group and time on CAP values. Estimates were adjusted for age, sex, and BMI.

https://doi.org/10.3390/nu18152476
